# A new macrofaunal limit in the deep biosphere revealed by extreme burrow depths in ancient sediments

**DOI:** 10.1038/s41598-017-18481-w

**Published:** 2018-01-10

**Authors:** S. L. Cobain, D. M. Hodgson, J. Peakall, P. B. Wignall, M. R. D. Cobain

**Affiliations:** 1 School of Earth and Environment, University of Leeds, Leeds, UK; 2 Ocean & Earth Science, University of Southampton, National Oceanography Centre (NOC), Southampton, UK

## Abstract

Macrofauna is known to inhabit the top few 10s cm of marine sediments, with rare burrows up to two metres below the seabed. Here, we provide evidence from deep-water Permian strata for a previously unrecognised habitat up to at least 8 metres below the sediment-water interface. Infaunal organisms exploited networks of forcibly injected sand below the seabed, forming living traces and reworking sediment. This is the first record that shows sediment injections are responsible for hosting macrofaunal life metres below the contemporaneous seabed. In addition, given the widespread occurrence of thick sandy successions that accumulate in deep-water settings, macrofauna living in the deep biosphere are likely much more prevalent than considered previously. These findings should influence future sampling strategies to better constrain the depth range of infaunal animals living in modern deep-sea sands. **One Sentence Summary:** The living depth of infaunal macrofauna is shown to reach at least 8 metres in new habitats associated with sand injections.

## Introduction

Deep-sea infauna is one of the most elusive branches of life on Earth; little is known about modern deep seabed environments, and less about the ancient. The limits of the macrofaunal biosphere in the deep-sea, and factors controlling life at depth below the seabed, are generally unknown.

In the modern, it is technologically challenging to collect undisturbed samples, and burrowing animals are usually found in marine sediments down to 20 cm^1^, or occasionally as deep as 2 m^2^. In ancient successions, the primary archive of deep zones of macrofaunal life is the ichnological (trace fossil) record in sedimentary rocks, which is limited by preservation factors with poor constraint on the original depth. Metre-scale (1–2 metre long) post-depositional burrows have been recorded on the base of turbidite sandstones, where individual depositional events are documented, providing depth control on infauna^3,4^. Rare cases of fauna extending beyond this depth require an open conduit in firm ground to allow filtration of seawater (e.g.^5–7^).

We have studied exhumed networks of clastic intrusions (injectites) produced by the injection of overpressured sand into surrounding strata (e.g.^8^). These injected sand dykes (vertical to sub vertical) and sills (horizontal) show evidence for post-injection living traces of macrofauna along their surfaces. Previously, injectites have been identified as favourable sites for colonisation by microbial life because they are permeable and provide a large sand-to-mud interface allowing for readily available electron donors and nutrients^9^. Here, we show that macrofauna also lived in injectites deep below the contemporaneous seabed.

## Geological background and dataset

**Figure 1:**
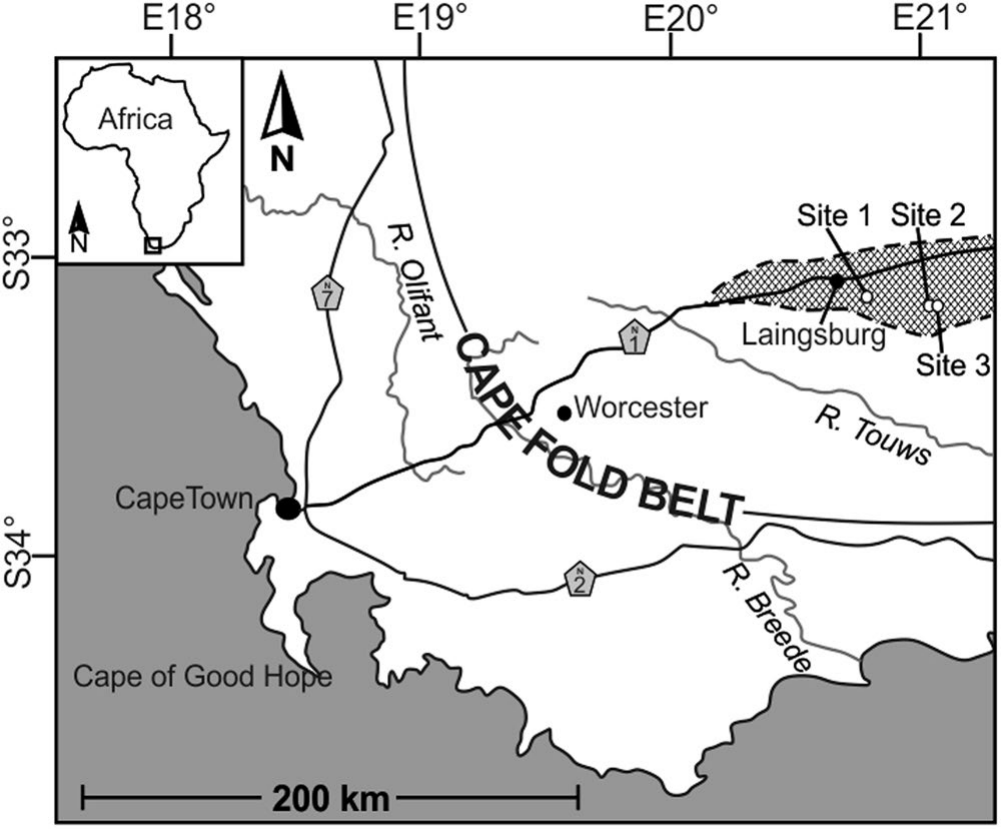
Location map of Laingsburg depocentre and outcrop sites 1–3, South Africa. Site 1 = Unit E, Geelbeck, Site 2 = Unit D, Slagtersfontein West, Site 3 = Unit D, Slagtersfontein East. Figure prepared in CorelDRAW × 8 (http://www.coreldraw.com/en/).

**Figure 2:**
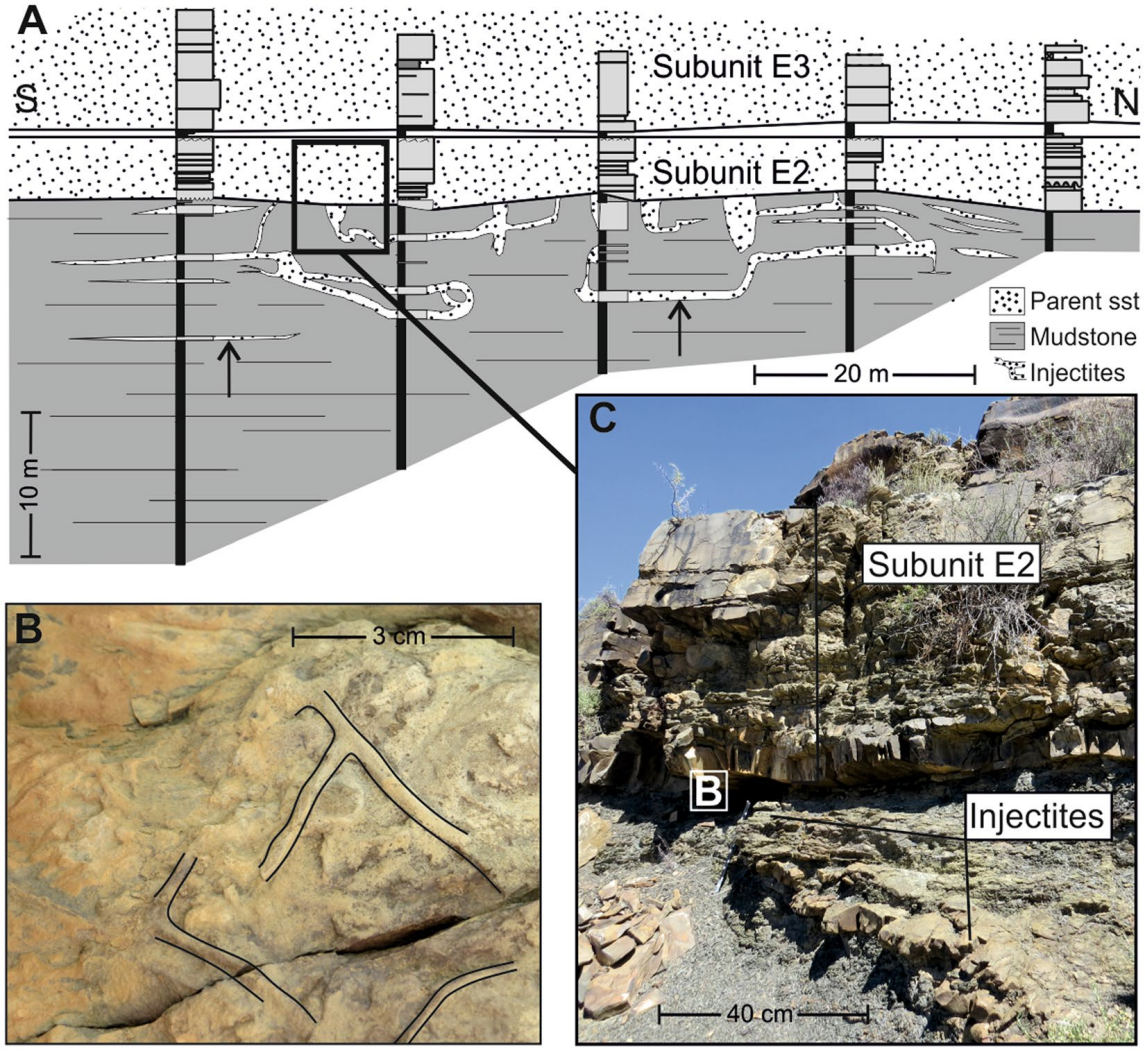
(**A**) A representative cross-section panel for outcrop sites with several stratigraphic logs taken from South to North. Here, Unit E at Geelbeck (Site 1) displays turbidite sandstones with underlying sandstone injections. Vertical to subvertical injectites are dykes, horizontal injectites are sills (see arrows). (**B**) Example of typical bioturbation seen on the base of Subunit E2 (see C). (**C**) Outcrop photograph demonstrating sharp and erosional contact between lobe sandstones (Subunit E2) and injectites below.

Three separate Permian outcrop sites from the SW Karoo Basin, South Africa (Fig. 1) exhibit sand injectites sourced from deep-marine turbidite sands in the Fort Brown Formation. Bioturbation is documented throughout this formation^10^ and ichnological assemblages include *Thalassinoides* and *Planolites* ^11^, commonly observed as hypichnia on sandstone bed bases (Fig. 2). At each site, the units have sharp and erosional bases (Fig. 2), and depositional architecture, and regional mapping demonstrates that they form stacked submarine lobe deposits^12,13^.

**Figure 3:**
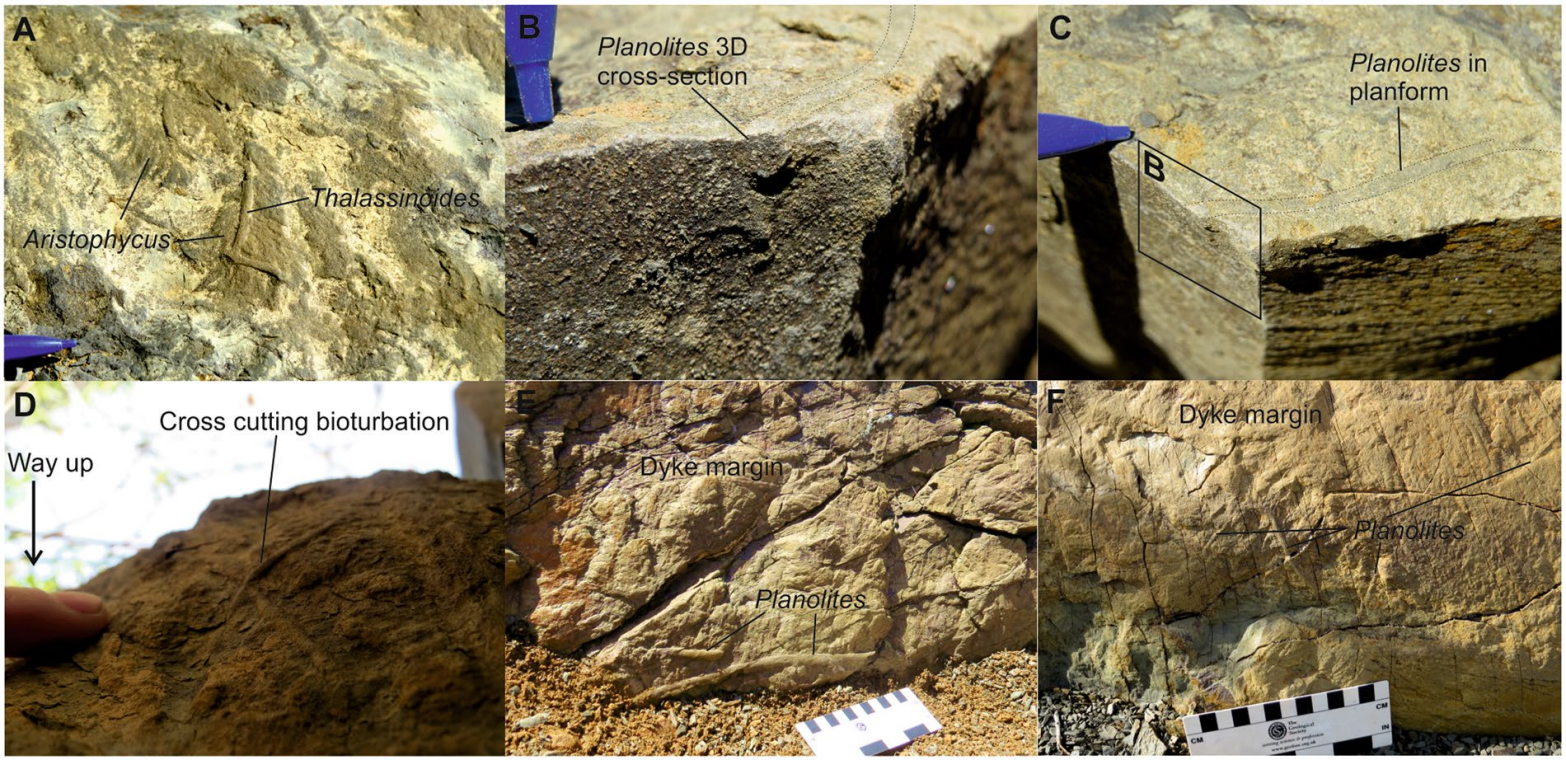
Typical examples of bioturbation found on clastic injectite margins. (**A**) Unit E (Site 1): dewatering structures (*Aristophycus*) on margin of a subvertical injectite, overprinted by *Thalassinoides* bioturbation. (**B**) Unit E (Site 1): *Planolites* tube protruding in cross-section of sill, planform of tube is outlined on the top margin of the sill. (**C**) view of (**B**) from alternate orientation. (**D**) Unit E (Site 1): Cross-cutting *Planolites* on base of sill. (**E**) Unit D (Site 2): Dyke margin with several examples of bioturbation, largest *Planolites* are indicated. (**F**) Unit C (Site 3): Dyke margin with several, smaller *Planolites*.

The injectites are sourced from fine sandstones, and are up to 0.5 m thick, sharp-sided, and propagated upwards and/or downwards and are discordant (dykes) or concordant (sills) to the stratigraphy (Fig. 2). A combination of 2 dimensional outcrops, a narrow grainsize-range and unvarying provenance ensures that specific source beds of the injectites cannot be discriminated. At sites 1, 2 and 3 (Fig. 1) injectites are found 8 m, 1.5 m and 3 m compacted depths respectively below overlying sandstones. The same trace fossils present on the base of sandstone beds throughout the Fort Brown Formation are observed on the margins of clastic injectites (Fig. 3), down to these lower limits.

### Interpretation

**Figure 4:**
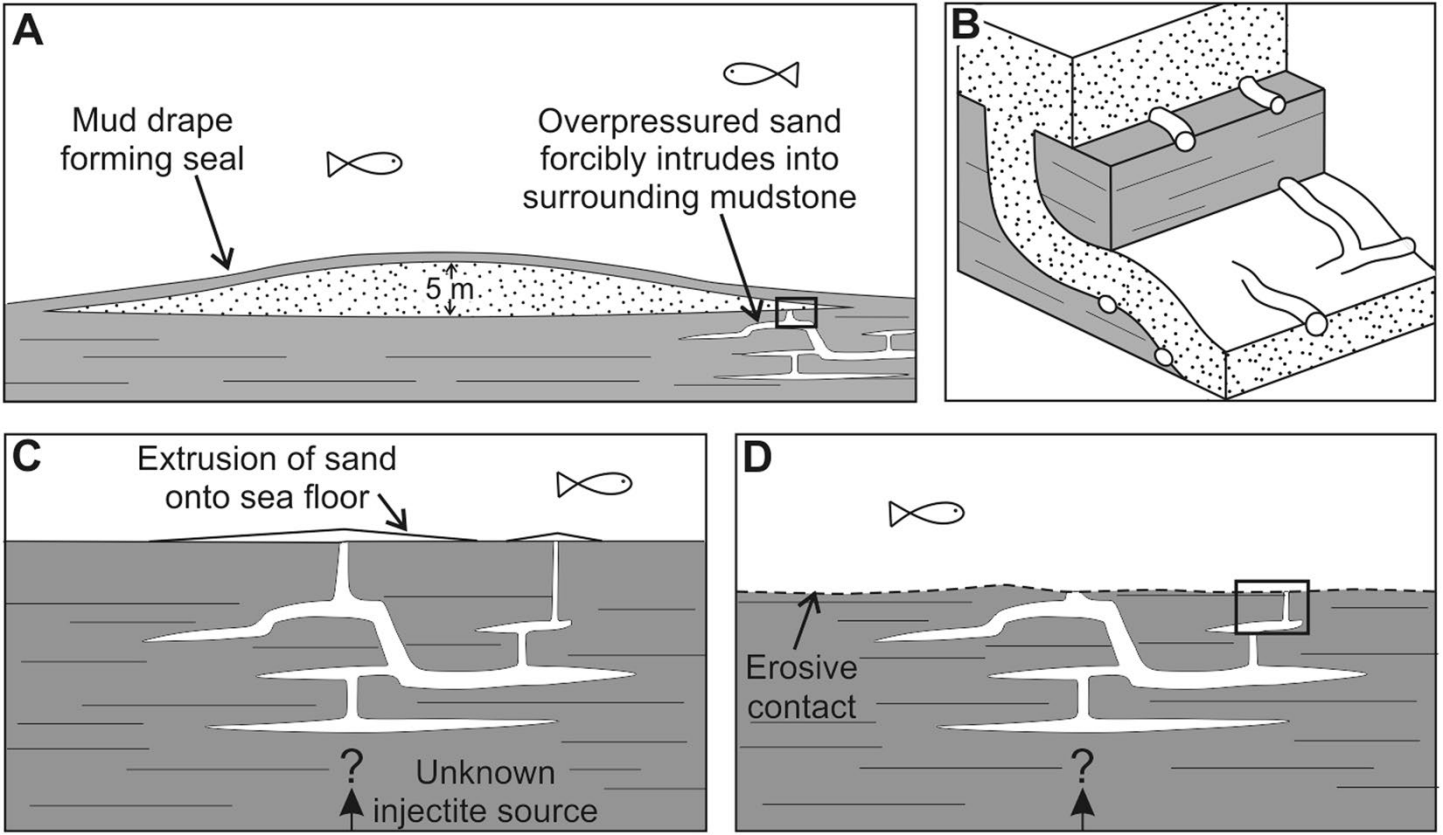
Different scenarios to explain presence of macrofaunal communities preservation in injectites. (**A**) Sandy lobe unit, with bioturbation along the sand-to-mud interface at the base (**B**), several metres below the seabed. Overpressure at the edge of the sandy lobes after rapid burial by muds causes unconsolidated sand to forcible intrude into underlying mud outwards from lobe centre. Macrofauna form new living traces on the sand-to-mud contacts that form the margins of the sand intrusions (**B**). Scenarios where a pre-existing injectite network either formed connection to the seabed through extrusion (**C**) or exhumation (**D**) to be exploited by macrofauna. Average lobe thickness in (**A**) based on^26^.

The structures on injectite margins are interpreted as trace fossils, and not grooves or markings formed through the injection process because they include the branching structure of *Thalassinoide*s and gently sinuous burrows of *Planolites* (Fig. 3A). Additionally, the structures show random orientations (Fig. 3D, F) fitting a bioturbation origin, whereas grooves would have a preferential direction caused by flow interacting with injectite-margins. It is clear that the bioturbation occurred after emplacement of the clastic injectites as it follows the sand-mud interface on both subvertical (dykes) and horizontal (sills) injectites (Fig. 3). If bioturbation on the injectites were casts of previously buried burrows then *Planolites* would be expected along sills only, parallel to stratal contacts. Traces in full relief are also observed on the top and bases of injectites, distinguishing them from seabed bioturbation, which will only have burrows in full relief on the lower side (Fig. 4B). In some cases, bioturbation overprints the dewatering structure *Aristophycus* (Fig. 3A), showing that clastic injection was followed by dewatering and then bioturbation. From these deductions, we can determine that *Planolites* and *Thalassinoides* formed after clastic injection. Forcible intrusion of sand into mud occurs in deep sea sediments in many sedimentary basins (e.g.^14^). Therefore, our discovery represents a widespread and unexplored macrofaunal environment. Previously, the organisms forming *Planolites* and *Thalassinoides* have been believed to have lived mainly in the top 20 cm of sediment, rarely reaching maximum depths of 1.5 m^3,15^. This limited depth range is due to the decline of oxygen and organic matter (food) in deeper levels of the sediment. Here, we document the presence of bioturbation on the margins of clastic injectites found at least 8 m below the seabed. In order to produce traces, organisms would need to survive long enough to burrow for hours-to-days. The size of the burrows (4–10 mm in diameter) suggests macro-infaunal invertebrates. We suggest 3 possible origins for the injected sand.

## Discussion

In the first scenario, injectites propagate downwards, sourced from overlying sands. This requires overpressure within the source sands in order to inject mixed sand and water downwards into the substrate. For overpressure to occur sands must be sealed by muds and there must be sufficient overburden (at least as thick as injectites are deep to promote downward injection). Turbidity currents deposit event beds that can be muddy^16^, occurring over hours to days, which could provide the necessary seal over a lobe for pressure to build during burial (Fig. 4A). In this case, macrofauna would have been living at several metres depth post-burial before being injected down with the flow into the substrate, whereupon they burrowed in this new setting. The concept of animals surviving both considerable transportation, and then living in oxygen-depleted environments on the seabed is well known (doomed pioneers;^17^). Alternatively, the fauna migrated down along the new network of sand, post injection.

In the second scenario, if injectites were sourced from below, then the host mudrock provides the seal required for overpressure and injection. Injectites could establish a seabed connection through extrusion^18^ allowing burrowers to then penetrate downwards (Fig. 4C). In the third scenario, a preexisting injectite network in the host mudrock, with a parent sand from below or above, is exhumed through seascape degradation (e.g.^19^). Organisms could then burrow downwards to exploit the injectite network as it acts as a source of new organic matter (Fig. 4D). The presence of thin overlying siltstones in the three examples (see supplementary text for detail), suggests that the lobes were not buried rapidly by mud-rich flows. These overlying siltstones, and the erosional basal contacts of the lobes, instead support exploitation by macrofauna of preexisting injectite networks through extrusion, exhumation, or a combination of both mechanisms.

In the above cases, for bioturbation to take place in the injectite *in situ*, the organisms would have had to survive i) potentially limited oxygen and POM (particulate organic matter) supply, ii) overburden pressures associated with injection depths, and if transported during injection iii) initial high energy transport. Life in a deep-marine environment often has a slow metabolic rate to survive cold temperatures and energy deprivation^20,21^. In the latter two scenarios above, it is possible for oxygen and POM to permeate the injectite network through connection to the seafloor. Where the injectite network is sealed, oxygen and nutrients are not replaced. Here, we model the possible survival time of organisms, parameterised by data based on contemporary polychaete populations, within an injectite as oxygen is depleted by respiration. We have estimated a time-frame which the organisms could survive within the injectite without replenishment of oxygen to give a potential life-span in a closed system using equation 1.

1 $$\frac{{\rm{d}}[{{\rm{O}}}_{2}]}{{\rm{d}}{\rm{t}}}=-\frac{[{{\rm{O}}}_{2}]}{{[{\rm{O}}}_{2{\rm{i}}}]}({\rm{S}}{\rm{C}}{\rm{O}}{\rm{C}}\times {{\rm{S}}}_{{\rm{b}}}+{\rm{N}}\times {{\rm{M}}}_{{\rm{r}}}\times {{\rm{S}}}_{{\rm{X}}})$$

where: t is time (days), [O_2_] is the dissolved oxygen at time t (ml/L), [O_2i_] is the initial (t = 0) dissolved oxygen concentration of the sediment (ml/L), SCOC is the Sediment Community Oxygen Consumption, which accounts for all the bacterial, meiofaunal and macrofaunal metabolic activity (mlO_2_/L/day), N is the abundance of polychaetes (number/L), M_r_ is the metabolic rate of polychaetes (mlO_2_/day), S_X_ is the proportion of survival of injected polychaetes and S_b_ the proportional survival of the background community.

Parameterisation using contemporary analogues (see Supplementary Materials) shows that macrofaunal organisms with a slow metabolism rate could potentially survive for up to 270 days with no replenishment of O_2_ (Fig. S3). If the injected sand was sourced from above, this period is required to overpressure the sand body by depositing muddy flows, inject, and produce the traces we observe. If sand is sourced from below, 270 days is the minimum time for oxygen depletion if surface burrow connections cannot be maintained, providing ample time for fauna to burrow downwards and exploit injected sand networks (Fig. 4).

Our study highlights a mismatch between observations taken of ancient and modern environments. Modern deep-sea biological studies target clays and silts as these are simpler to sample. Standard sampling methodologies, such as piston coring, are typically unable to sample sandy sediments due to lack of cohesion of the grains. In contrast, rare examples of deep tier bioturbation from the rock record have been able to demonstrate that burrowing occurred in deep-marine sands at 1–2 metres below the contemporaneous seabed^3,4,22^. Here we demonstrate burrowing to much greater depths, up to an uncompacted depth of 8 metres. The high degree of compaction of fine-grained sediments at such depth is likely to make burrowing activity unlikely whereas the deep sand burrowing we document here suggests such activity may be common-place (but unsampled in the present day).

Whilst we have demonstrated in these examples that macrofauna were present in the deep biosphere, a key question is whether these are exceptional cases related to unique conditions, or whether the conditions conducive to burrowing of macrofauna to multi-metre depths are widespread. Sand-rich sedimentary environments, in the form of lobe deposits and channel-fills are present over very large portions of submarine fans (e.g.^12,23–26^), themselves the largest sedimentary deposits on Earth^27^. Macrofauna might be expected to burrow to depth in many of these sub-environments, however, the present examples show unique evidence that the depth reached is preserved. In most cases, the macrofauna will be preserved in the rock record but the information on their depth below the contemporaneous seabed is lost. More recently, there has been a rapidly growing recognition of sand injection in many deep-marine settings, from channels on the slope^28^ to lobes on the basin floor^29,30^. Given the ubiquity and thickness of sand-rich sedimentary successions in many deep-sea environments, and the widespread occurrence of sediment injection then it would seem probable that such macrofauna in the deep biosphere are more prevalent than currently recognised.

## Conclusions

Our findings have several biological and geological implications, i) unusually, we can quantify a minimum depth below the seabed that organisms inhabited in ancient sediments, ii) show that the deepest organisms may be present in sandy sediments, rather than the clays and silts typically targeted in modern seabed investigations, iii) show that less organics are preserved due to carbon consumption during metabolic activity, which then also changes the sediment fabric at depth, with grains being processed and sorted into burrow structures, and iv) most importantly, we have shown that macrofaunal life survives for periods living at depths of up to 8 m below the seabed, giving an entirely new (lower) limit to the macrofaunal biosphere. This new evidence of bioturbation in Permian sandstones at many metres below the seabed suggests that we need to adapt sampling strategies when looking for macrofaunal life in the deep biosphere. Targeting sandy successions in deep-marine systems offers potential for observing the behaviour and diversity of organisms at greater depths than has so far been hitherto appreciated in modern deep-sea sediments.

### Data availability

All data generated or analysed during this study are included in this published article (and its Supplementary information files)^31–41^.

## Electronic supplementary material

Supplementary information
