# Supplementary material for: A new macrofaunal limit in the deep biosphere revealed by extreme burrow depths in ancient sediments
Source: Sci Rep. 2018 Jan 10;8:261. doi: 10.1038/s41598-017-18481-w (PMC5762628; doi:10.1038/s41598-017-18481-w)
Supplement: Supplementary file 1 — Supplementary information [file 41598_2017_18481_MOESM1_ESM.pdf]

Supplementary Materials for

A new macrofaunal limit in the deep biosphere revealed by extreme burrow depths in ancient sediments

S.L. Cobain, D.M. Hodgson, J. Peakall, P.B. Wignall, M.R.D. Cobain

Correspondence to: sarah.cobain@pds.group

This PDF file includes:

Supplementary Text and Data
Figs. S1 to S4
Reference list

Supplementary Text and Data

Geological setting

The Permian Ecca Group, a succession of siliciclastic material, was deposited within the Tanqua and Laingsburg depocentres of the SW Karoo Basin [31]. The Laingsburg depocentre (Fig. 1) comprises a shallowing upward stratigraphic succession. This study focuses on outcrops of the Fort Brown Formation; a 400 m thick submarine slope succession [31, 32] (Fig. S1). The Fort Brown Formation comprises sand-prone lithostratigraphic units C to G, which are further divided into subunits by laterally extensively thin (<2 m thick) fine siltstones consistent with palaeogeographic changes across a unit [12, 33]. Each unit is separated by a thick regionally extensive mudstone (siltstone and claystone) (>10 m thick) interpreted to represent a basin-wide shut down in sand supply [31, 32].

Bioturbation has been documented throughout the Fort Brown Formation [10, 11, 13]. Small burrows occur within mudstone beds that separate sand beds [11], in thin-bedded siltstones [13] and on the base of structureless and rippled sandstones [11]. Ichnofacies assemblages are interpreted to be primarily *Chondrites* and *Planolites* [11].

Individual outcrop sites are indicated by place names and stratal units (Figs. 1 and S1). At each site, the units are submarine lobe deposits [12, 13] and comprise thin, very fine sandstones capped by mudstones (Fig. 3A and 3C). Clastic injectites are recognised through dykes cross-cutting stratigraphy, sills stepping up and down stratigraphy, and

their sharp sided nature on top and base margins [28, 29]. In the Karoo Basin, injectites are sourced from the very fine sandstone units, they are up to 0.5 m thick, sharp sided, and usually subvertical. At sites 1, 2 and 3 the injectites are 8 m, 1.5 m and 3 m compacted depths respectively, below the capping sand. The same trace fossils present on the base of units (Fig. 2B) are also observed on the margins of clastic injectites down to their lowermost occurrence (Fig. 3).

Outcrop observations

Site 1: Unit E, Geelbeck

At Geelbeck, Subunit E1 is absent, Subunit E2 is an intraslope lobe complex comprising three stacked lobe deposits, the lowermost of which was deposited in a highly-confined environment [13]. The base of Subunit E2 is very well exposed, and therefore so are examples of *Planolites* (Fig. 2); individual borrows are up to 10 cm long and <1 cm in width. The basal beds of Subunit E2 onlap an erosional surface and mainly comprise structureless sandstones, with ripples present towards the bed tops.

The dykes directly in contact with the base of Subunit E2 form an abruptly downward tapering or upward opening cone ~1 m in width and 1.5 m in depth (Fig. 2C). Each cone-like feature comprises multiple dykes passing into or from a single dyke 5-20 cm wide that are connected to multiple sills and dykes below. Sills do not step through stratigraphy and individually extend up to 20 m. On the margins of the clastic injectites, the same trace fossil, *Planolites*, is present (Figs. 3B-3F) that occurs on the base of

Subunit E2 (Fig. 2B). Additionally, on a subvertical dyke ~2 m below the base of Subunit E2 are dewatering structures (*Aristophycus*) (Fig. 3A), that are overprinted by *Planolites*. The bioturbation is present on tops and bases of sills and on the margins of dykes and is observed at least 8 m (compacted thickness) stratigraphically below Subunit E2, which is the extent of the injectites.

Site 2: Unit D, Slagtersfontein West

Unit D at Slagtersfontein West is represented by the lowermost sequence, Subunit D1. Subunit D1 is interpreted to be lowstand lobe deposits [12] similar in character to those described by [34]. Where observed, Subunit D1 has a sharp, sometimes erosive, laterally extensive basal contact with the underlying mudstone unit and comprises structureless sand. The subunit is locally deeply incised by a younger submarine channel surface [35]. Injectites are observed directly below the base of Subunit D1 as <40 cm thick dykes, which penetrate at least 2 m of the underlying mudstone (Fig. S2B). The margins of these dykes exhibit randomly orientated, and up to 20 cm long, *Planolites* (Fig. S2B).

Site 3: Unit C, Slagtersfontein East

Here, C2 is the only subunit of Unit C present and is interpreted to be the proximal edge of a lobe complex [12]. Locally, the basal surface of Subunit C2 cuts into the underlying B/C mudstone with the lower beds consisting of structureless and amalgamated sandstones. *Planolites* is present along the base of Subunit C2. Injectites exposed in the Slagtersfontein area are primarily hosted within the regional mudstone separating Units

B and C (Fig. S2A). The majority of injectites at the Slagtersfontein outcrop are 0.1–0.6 m thick sills that extend laterally for up to 500 m. These connect to subvertical dykes directly in contact with the base of Subunit C2. *Planolites* is present on the top and base of sills and dykes up to 2 m stratigraphically below C2. The burrows are randomly orientated and common across the injectites.

Outcrop summary

In each of the cases presented here, clastic injectites are hosted within mudstones, and there is a direct connection between the injectites and the overlying capping sandstones. The grain-size is too narrow and provenance too consistent in the sandstone of the Fort Brown Formation to be used to discriminate the source of the injectites. Furthermore, two-dimensional outcrops make it difficult to determine if the sands were injected from above or below since a deeper connection may be out of the plane of the section. In all cases, there is evidence for erosion on the surface between the capping sands and the underlying mudrock that hosts the injectites. Given this, and the thin and silty nature of the overlying fine-grained units that suggest that the lobe sands were not rapidly buried by mud-rich flows, the scenario where the injectite networks existed prior to exploitation by macrofauna appears more likely. However, we cannot discriminate whether the injectites were sourced from below and connected to the seabed to form extrudites, or were exhumed through submarine erosion with an underlying or overlying source, although an underlying parent sand is considered more likely given pressure gradients and depth of erosion required.

Model of oxygen consumption in injected sediments

The first scenario presented assumes no replenishment of oxygen and POM (particulate organic matter), therefore it is necessary to calculate the amount of time macrofauna such as polychaetes could survive if oxygen and POM were suddenly shut off, i.e. if life were living in an injectite where no new source of pore water is present. In the latter two scenarios presented, there is potential for oxygen and POM to permeate through or along the margins of the injectite network, or through burrow irrigation. Therefore, in these two situations, this model of oxygen consumption could present a minimum time of oxygen and POM depletion.

Here we consider that not all life that is present on the seabed will exploit the injectite networks; if the macrofauna are forcibly intruded down then some may not survive. Alternatively, if macrofauna are migrating down post injectite emplacement, not all will actively do so, with many remaining on the seabed. Here we explicitly parameterise the model based on polychaete data as these are some of the most widely studied burrowing deep-sea invertebrates, however results will be similar (of the same order of magnitude) for other phyla – see fig. S4.

The numerical model for solving dissolved oxygen concentrations is given as:

$$\frac{dO_2}{dt} = -\frac{O_2}{O_{2\,i}}(SCOC \times S_b + N \times M_r \times S_x) \qquad \text{eq. 1}$$

where:

- t – Time (days, time steps of 0.01)
- O_2 – Dissolved oxygen (at time t) (ml / L)
- $O_{2\,i}$ – The initial (t = 0) dissolved oxygen concentration of the sediment (ml / L)
- $SCOC$ - The Sediment Community Oxygen Consumption, which accounts for all the chemical, bacterial, microfaunal and macrofaunal metabolic activity (mlO_2 /L / day)
- N – Abundance of polychaetes (number / L)
- M_r - Metabolic rate of polychaetes (mlO_2 / day)
- S_x – Proportion of survival of injected polychaetes (unitless, ratio from 0 - 1)
- S_b – Proportional survival of background community

Model assumptions:

Several assumptions were made in the building and use of the above equation: i) oxygen will be the main limiting factor for metabolism, as there are internal stores of carbon. Low levels of oxygen are known to have substantial modifying effects to macrobenthic communities in the deep sea such as in Oxygen Minimum Zones (OMZs) [36]. ii) Pore waters and sediments become homogenised through injection, such that everything is well mixed and any pre-injection structure in fauna or oxygen levels is lost and no re-structuring occurs post injection. iii) There are no external sources of input of oxygen once the parent body is sealed and injection has occurred. iv) Population dynamics are on too long a time-scale post injection, and so are not considered. v) Rates of oxygen uptake decrease as surrounding oxygen concentration decreases, through the process

of passive diffusion across biological and chemical surfaces - oxygen uptake rates are proportional to the current oxygen concentration (scaling term $\frac{O_2}{O_{2\,i}}$).

Model parameterisation:

$O_{2\,i}$

A review by [36] on the effects of oxygen on deep sea sediment communities reports values of 0.2 – 6.21 ml / L from various ocean basins for the concentration of oxygen in waters at the sediment surface in deep sea environments. For a water depth of 740 m, [12] recorded near bottom oxygen concentration values as low as 0.08 ml / L in the eastern Pacific in an oxygen minimum zone (OMZ). Typically, deep water has values near the saturation value, except for OMZs, with a gradual decrease as they progress further from the site of origin due to metabolic processes, down to 3.6 ml / L in the eastern Pacific [37]. For the initial oxygen concentration, we used a value of 8 ml / L reflecting relatively well-oxygenated water overlying seafloor sediments. We assume the site is not in an OMZ. To correct for inclusion of sandy sediment in the volumetric space, we assume a porosity (Φ) of 46% such that:

$$O_{2\,i} = O_{sw} \times \phi \qquad \text{eq. 2}$$

This is typical of the sediment injected, fine to very fine sands.

SCOC

All the biologic activity within the sediment is accounted for by the sediment community oxygen consumption (SCOC). This includes macrofauna, however, they typically only

account for a small portion of the SCOC, whereas in the model presented, we add an additional polychaete population. [38] measured values of total oxygen uptake in sediments ranging from 0.0403 – 0.347 $mlO_2L^{-1}day^{-1}$ in the south east Atlantic. They suggest these are higher than typical values elsewhere due to high surface productivity. [39] measured median value in sediments east of Svalbard, of 0.0618 $mlO_2L^{-1}day^{-1}$. An average presented by [40] for the north Atlantic and Pacific from depths of between 1 and 2 km is 0.0508 $mlO_2L^{-1}day^{-1}$. Here, we used the median value of 0.0618 $mlO_2L^{-1}day^{-1}$, since this included sampling from sandy sediments, at high latitude, more analogous to the deposits in the Karoo Basin.

N

Samples from the Rockall Trough (West of Scotland) found an average of 0.39 - 1.724L^{-1} depending on the mesh size used to sort sediments [40]. Whereas [41] found macrofauna abundances of around 1.8 L^{-1}. We used a value of 2 L^{-1}.

M_r

Metabolism of deep-sea organisms scales in a similar way to shallow water species, where size and temperature account for most of the variability [21]. [20] empirically showed that the respiration rates of deep sea organisms (taken from areas of 2-4°C) scales with their weight such that:

$$R = 7.4 \times 10^{-3} \times W^{-0.24} \quad \text{eq. 3}$$

where *R* is the respiration rate (per day) and W is the weight (in mgC) of the organism. We modified this equation to give the respiration rate in units of $ml0_2day^{-1}$ such that:

$$M_r = \frac{1}{0.44} \times 7.4 \times 10^{-3} \times W^{0.76} \quad \text{eq. 4}$$

where 1/0.44 is the mobilisation of oxygen (in ml) per mg of carbon (taken from [20].

The weight used was 0.428 mgC, the average size for the deep sea macrofauna used by [20] in their study of nematodes, copepods and polychaetes.

$\mathbf{S_x}$

As data were unavailable, a value of 0.5 was used, i.e. only half the population of polychaetes survive the injection (or actively colonize the injectite) to exploit the injectite network post-emplacement.

$\mathbf{S_b}$

As data were unavailable, a value of 0.5 was used. Mechanical shaking of the sediments causes microfauna to be lost as they typically have lower densities than sediments. Further, some proportion of reduced chemicals in the sediment will be oxidised as mixing with overlying waters occurs, reducing oxygen uptake by chemical means post-injection.

Unit Conversions and results:

Units reported in the literature needed to be converted in many cases prior to being input into the model. In the literature, SCOC and abundances are typically reported as per unit area of sediment surface, therefore these have been converted to volume to provide a depth aspect on oxygen consumption within a community by assuming a depth of 1 m. Molar oxygen concentrations were converted to ml / L using the ratio of 1 mlO_2 / L seawater = 44.661$\mu molO_2$ / L (from ICES oceanography).

Below an oxygen concentration of 0.45 ml / L, the community structure of deep-sea macrofauna becomes adversely affected, however it appears that polychaetes are the most tolerant of macrofaunal taxa [36]. We therefore took the threshold of polychaetes to be 0.2 mlO_2 / L. A length of time can therefore be estimated, before oxygen becomes too low in the sediment. As parameterised above, this occurs at 269.8 days, which provides time for burial and sealing by mud-rich turbidity currents and injection to occur (Scenario 1), or, for macrofauna to exploit injectites and travel down to their lower most occurrence (Scenario 2 and 3) (Fig. S3).

An analytical sensitivity analysis of the model is shown in Fig. S4, and demonstrates that the model is relatively insensitive to the precise parameters used, and consequently there would have been sufficient time for organisms to have produced the observed burrows.

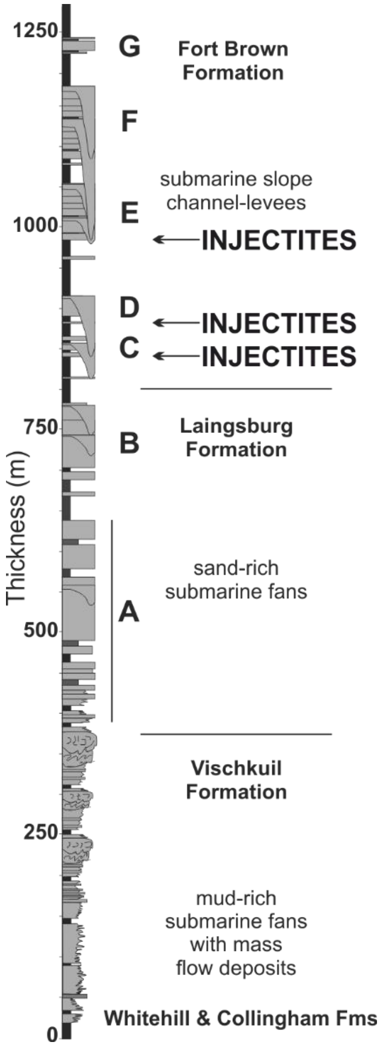

Fig. S1. Summary stratigraphic log of Laingsburg depocentre, letters A-G refer to Units A-G [31].

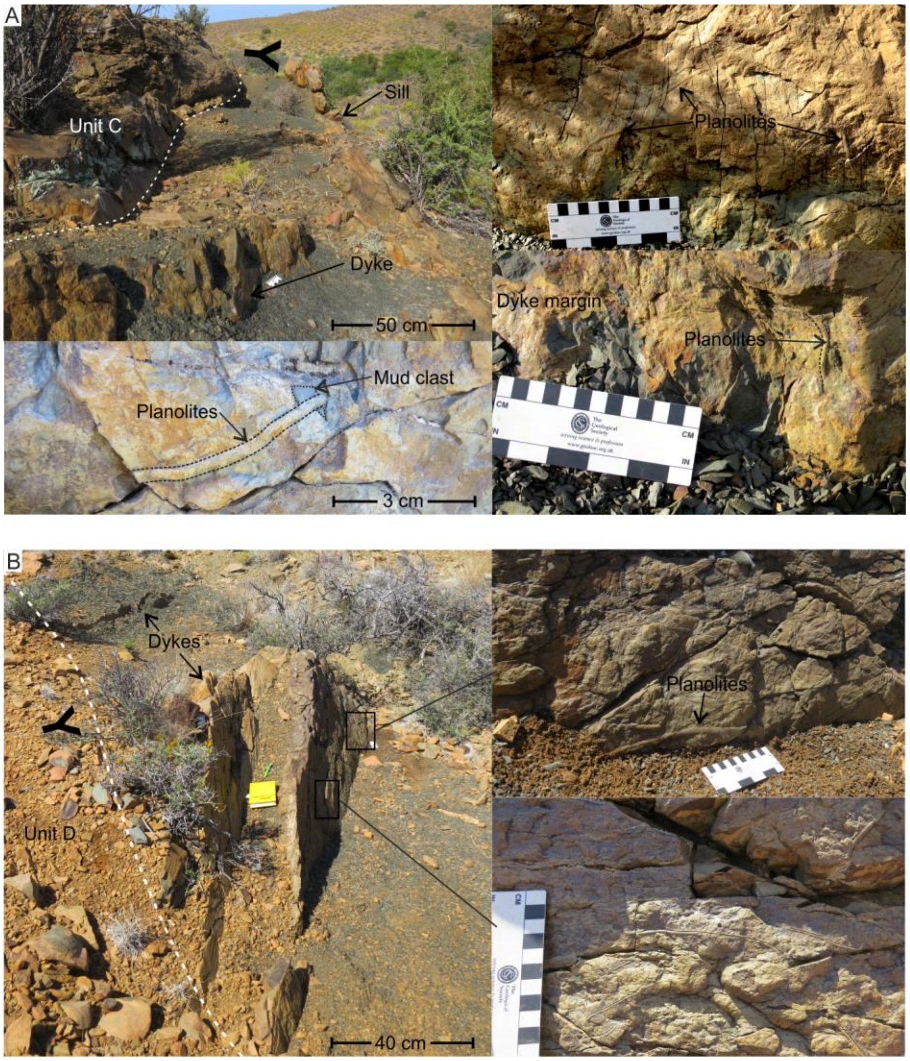

Fig. S2. More bioturbation examples from injectites related to Units C and D. A) Unit C, Slagtersfontein East outcrop. Images depict relationship of injectites to base of unit and examples of bioturbation along both dykes and sills. B) Unit D, Slagtersfontein West. Dykes in contact with base of Unit, and bioturbation examples from dyke margins.

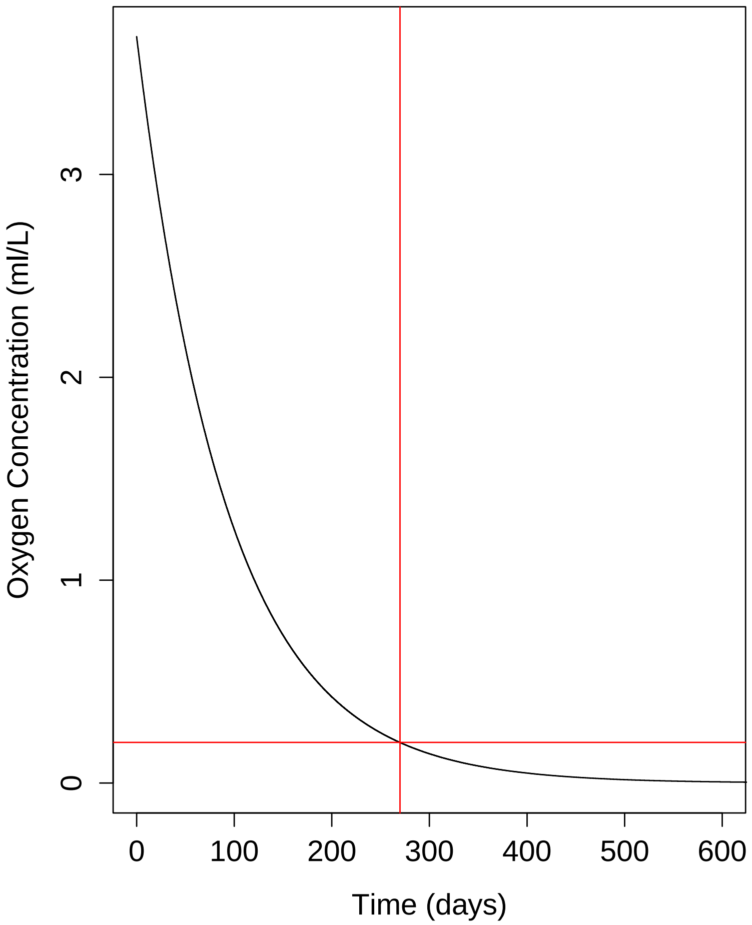

Fig. S3. Oxygen depletion graph. Rate of oxygen depletion within the sediment starting from the point of burial (and therefore isolation), or from initial point of colonisation of an exhumed / extruded network, as calculated from the numerical model. Horizontal red line indicates minimum O_2 needed for survival, vertical red line is the cutoff of life in time according to O_2 concentration.

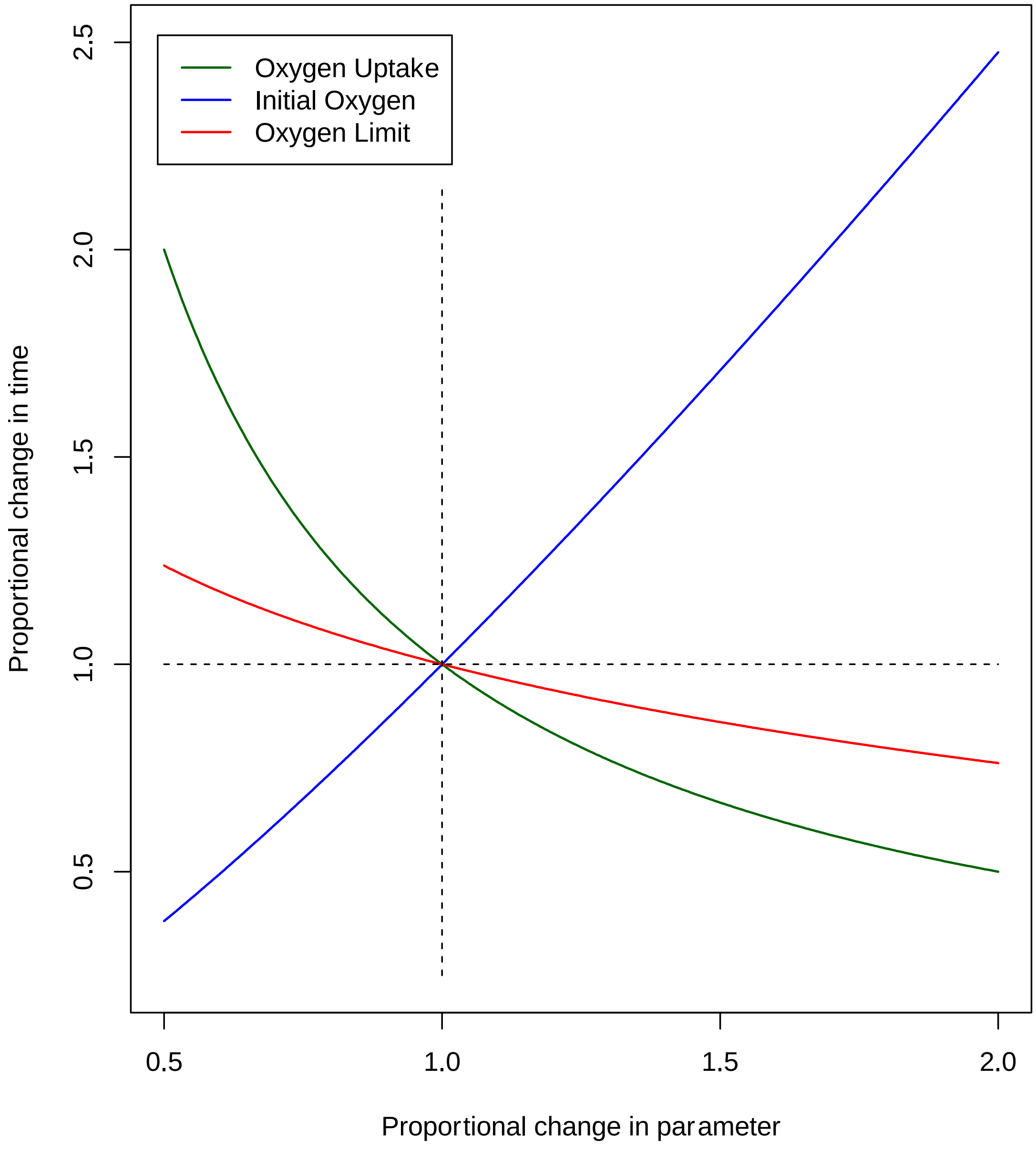

Fig. S4. Graph showing proportional change in time until oxygen limit is reached due to proportional change in model parameters. Oxygen uptake is the combined sources of oxygen depletion within the sediment, initial oxygen is the starting oxygen concentration within the sediment and the oxygen limit is the minimum oxygen concentration prior to

detrimental effects to the polychaete population. Dashed lines show intersection of model parameters with no change.

References

10. Oliveira, C. M., Hodgson, D. M. & Flint, S. S. Distribution of soft-sediment deformation structures in clinoform successions of the Permian Ecca Group, Karoo Basin, South Africa. *Sed. Geol*. **235,** 314-330 (2011).

11. Morris, E. A. *et al*. Sedimentology, stratigraphic architecture, and depositional context of submarine frontal-lobe complexes. *J. Sed. Res.* **84,** 763-780 (2014).

12. Van de Merwe, W. C., Hodgson, D. M., Brunt, R. L. & Flint, S. S. Depositional architecture of sand-attached and sand-detached channel-lobe transition zones on an exhumed stepped slope mapped over a 2500 km^2 area. *Geosphere* **10,** 1076-1093 (2014).

13. Spychala, Y. T., Hodgson, D. M., Flint, S. S. & Mountney, N. P. Constraining the sedimentology and stratigraphy of submarine intraslope lobe deposits using exhumed examples from the Karoo Basin, South Africa. *Sed. Geol*. **322,** 67-81 (2015).

20. Mahaut, M. Sibuet, M. & Shirayama, Y. Weight-dependent respiration rates in deep-sea organisms. *Deep-Sea Res. Pt I: Oceanographic Research Papers* **42,** 1575-1582 (1995).

21. McClain, C. R., Allen, A. P., Tittensor, D. P. & Rex, M. A. Energetics of life on the deep seafloor. *PNAS* **109,** 15366-15371 (2012).

28. Hurst, A., Scott, A. & Vigorito, M. Physical characteristics of sand injectites. *Earth-Sci. Rev*. **106,** 215–246 (2011).

29. Cobain, S. L., Peakall, J. & Hodgson, D. M. Indicators of propagation direction and relative depth in clastic injectites: Implications for laminar versus turbulent flow processes. *Geol. Soc. Am. Bull.* **127,** 1816-1830 (2015).

31. Flint, S. S. *et al*. Depositional architecture and sequence stratigraphy of the Karoo basin floor to shelf edge succession, Laingsburg depocenter, South Africa. *Mar. Pet. Geol*. **28,** 658-674 (2011).

32. Di Celma, C. N., Brunt, R. L., Hodgson, D. M., Flint, S. S. & Kavanagh J. P. Spatial and temporal evolution of a Permian submarine slope channel–levee system, Karoo Basin, South Africa. *J. Sed. Res*. **81,** 579-599 (2011).

33. Figueiredo, J. J., Hodgson, D. M., Flint, S. S. & Kavanagh, J. P. Depositional environments and sequence stratigraphy of an exhumed Permian mudstone-dominated submarine slope succession, Karoo Basin, South Africa. *J. Sed. Res*. **80,** 97-118 (2010).

34. Prélat, A. & Hodgson, D. M. The full range of turbidite bed thickness patterns in submarine lobes: controls and implications. *J. Geol. Soc. London* **170,** 209-214 (2013).

35. Hodgson, D. M., Kane, I. A., Flint, S. S., Brunt, R. L. & Ortiz-Karpf, A. Time-transgressive confinement on the slope and the progradation of basin-floor fans: Implications for the sequence stratigraphy of deep-water deposits, *J. Sed. Res.* **86,** 73-86 (2016).

36. Levin, L. A. & Gage, J. D. Relationships between oxygen, organic matter and the diversity of the bathyal macrofauna. *Deep-Sea Res. II* **45,** 129-163 (1998).

37. Gage, J. D. & Tyler, P. A. Deep-sea biology: A natural history of organisms at the deep-sea floor. *Cambridge, UK: Cambridge University Press* (1991).

38. Glud, R. N., Gundersen, J. K., Jørgensen, B. B., Revsbech, N. P. & Schulz, H. D. Diffusive and total oxygen uptake of deep-sea sediments in the eastern South Atlantic Ocean: *in situ* and laboratory measurements. *Deep-Sea Res. I* **41,** 1767-1788 (1994).

39. Piepenburg, D. *et al*. Partitioning of benthic community respiration in the Arctic (northwestern Barents Sea). *Mar. Ecol. Prog. Ser.* **118,** 199-213 (1995).

40. Gage, J. D., Hughes, D. J. & Gonzalez Vecino, J. L. Sieve size influence in estimating biomass, abundance and diversity in samples of deep-sea macrobenthos. *Mar. Ecol. Prog. Ser.* **225,** 97-107 (2002).

41. Levin, L. A., Huggett, C. L. & Wishner, K. F. Control of deep-sea benthic community structure by oxygen and organic-matter gradients in the eastern Pacific Ocean. *J. Mar. Res.* **49,** 763-800 (1991).
